# Detection of reed using cnn method and analysis of the dry reed (*phragmites australis*) for a sustainable lake area

**DOI:** 10.1186/s13007-023-01042-w

**Published:** 2023-06-24

**Authors:** Cristian Dragos Obreja, Daniela Laura Buruiana, Elena Mereuta, Alina Muresan, Alina Mihaela Ceoromila, Viorica Ghisman, Roxana Elena Axente

**Affiliations:** 1 Faculty of Engineering, Interdisciplinary Research Centre in the Field of Eco-Nano Technology and Advance Materials CC-ITI, “Dunarea de Jos” University of Galati, Galati, Romania; 2 Department of Mechanical Engineering, “Dunarea de Jos” University of Galati, 47 Domneasca, 800008 Galati, Romania; 3 Research and Development Center for Thermoset Matrix Composites, Cross-Border Faculty, , “Dunarea de Jos” University of Galati, Galati, Romania; 4 Medicine and Pharmacy Faculty, “Dunarea de Jos” University of Galati, 47 Domneasca, 800008 Galati, Romania

**Keywords:** Biomass, Reed, Convolutional neural networks, Sustainable

## Abstract

**Background:**

Common reed (Phragmites australis L.) is a highly productive wetland plant and a possible valuable resource of renewable biomass worldwide. For a sustainable management the exploitation of reed is beneficial because the increasing demand for sustainable biomass which presents reed bed areas and wetlands. Knowing the properties of plant biomass obtained from reeds is essential both for the effect on combustion equipment and for the impact on the environment. Brates Lake, situated in Galati, Romania is a natural watershed with reed plantations.

**Results:**

We used the convolutional neural network method combined with the cropped image techniques represent a powerful tool for high-precision image-based biomass detection in lake areas. The study aimed to investigate the morphological and chemical parameters through SEM–EDX analysis and pH, conductivity, nitrate anion, nitrite anion, total nitrogen, sulphate anion, sulphide anion, phosphate anion concentrations were determined from reed extract. The samples have a moderately acidic reaction pH 4.91–4.98. The number of soluble salts in the reed extract is in the range of 3.24–4.70 g/L, the values are within normal limits, providing the plant with the necessary nutrients.

**Conclusions:**

This is the first time that neural networks are used for the detection and prediction of areas at risk for biodiversity (reduction of water gloss until it disappears, imbalances caused by keeping reeds dry in water) caused by the aggressive and uncontrolled growth of reeds.

**Supplementary Information:**

The online version contains supplementary material available at 10.1186/s13007-023-01042-w.

## Background

Reed (Phragmites) is one of the most widespread wetland plants in Europe, the Middle East and America [1]. Wet sites are the characteristic of reed *Phragmites australis* which grows most often at the borders of gulfs and lakes, throughout on the banks of river and on nutrient-rich peatbogs [2]. The common reed *Phragmites australis* is a species of energy plant, used as a basis for biomass to obtain energy from primary waste. Thus, reed is used as an energy source in European countries such as Estonia, Finland, The Netherlands, Hungary and Romania. Knowing the properties of plant biomass obtained from reeds is essential both for the effect on combustion equipment and for the impact on the environment. The reed biomass is harvested in winter because of the compatibility of various applications and, to minimize the conflict with nature conservation. From the point of view of the economic perspective the exploitation of reed is beneficial for a sustainable management, furthermore that for a circular bioeconomy has become attractive the increasing demand for sustainable biomass which presents reed bed areas and wetlands [3, 4]. The utilization of reed as an industrial material for thatching production is suitable naturally dried winter harvested reed long, straight, and flexible stems with length 1.5–2.3 m and diameter 3–12 mm [5, 6]. Reed stems can be utilized as insulation material for walls and roof coverings by combining a high volume-to-weight ratio with high air content which supports a good indoor climate [7]. In the past, reed biomass was used as reed pulp and paper due the fact that the cellulose content of reed is ranging between 33 and 59% [8]. Also, the reed can be used as a source of polymer, as raw material to separate out the hemicelluloses, lignin and cellulose which are then used in other applications [9]. *P. australis* an important component that can contribute to depollution processes owed to its possibility to absorb mineral ions through roots at very low concentrations, making this process very efficient [10]. Catana et al. [11] screened the elements with different potentials (critical raw materials—CRMs; toxic; potentially toxic) from Phragmites australis leaves along the Colentina urban river and showed that the values of the elements in the anthropogenic source were different from the periurban and urban ones.

The reed used in this study was collected from Brates Lake, Galati, Romania. Brates Lake, situated in South-East of Romania, was declared a bird-faunistic special protection area by Government Decision no. 971 (2011) (for the amendment and completion of Government Decision no. 1284/2007 regarding the declaration of areas of special avifaunistic protection as an integral part of the European Natura 2000 ecological network in Romania) [12] and covers an area of 15,681.70 ha. It overlaps with the protected areas: the Danube Delta (biosphere reserve) and the Lower Prut Low Meadow Natural Park.

The aim of the study was to detect using artificial intelligence and to analyse the structural and chemical parameters of the dry reed for their importance regarding the growth and development of the plant and to monitor the values that exceed the accepted norms, because it will cause the pollution of the waters where the reeds grow.

Here we show for the first time the detection and prediction using convolutional neural networks at areas of risk for biodiversity (reduction of water gloss until it disappears, imbalances caused by keeping reeds dry in water) caused by the aggressive and uncontrolled growth of reeds.

## Materials and methods

### Area description and sampling

**Fig. 1:**
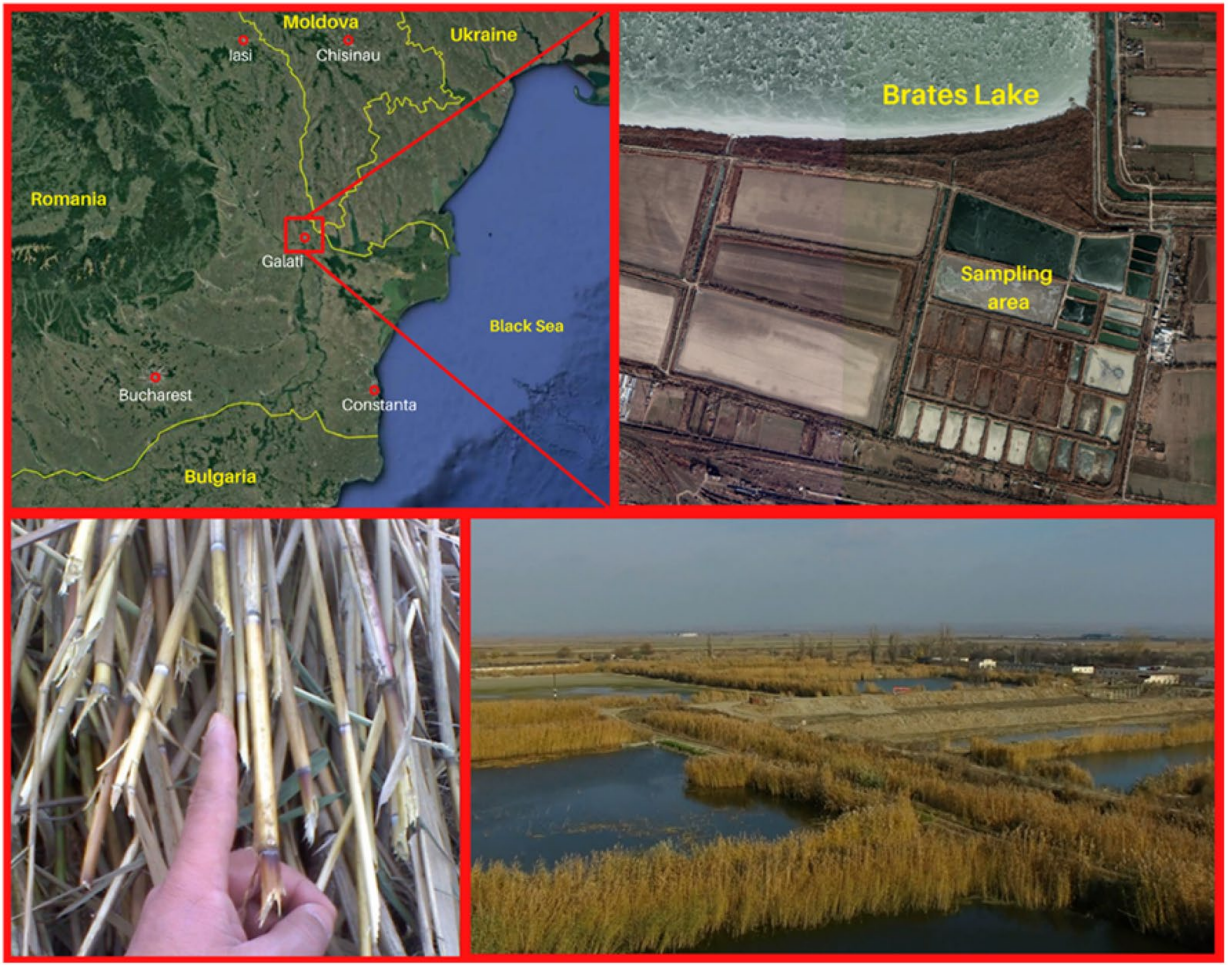
Map of the sampling site location

The reed specimens type Phragmites australis L. were analysed microstructurally (SEM analysis) and chemically (EDX analysis). For this study the reed samples were collected from Brates Lake, Galati, Romania (Fig. 1) and the analysed area was the stem of the reed.

The photos were taken in autumn (November 2022) because the sampling area belongs to bird-faunistic special protection area.

Reed, which is a hydrophytic annual plant (adapted to the aquatic environment), requires a specific protocol for fixing and preparing the sample for microscopic study. For these analyses, 3 types of samples were prepared in this way, 1 control reed samples and 2 reed samples that were immersed and kept in water for 14 days (100 g of reed per 500 mL of water). Prior to the analysis, the 3 types of samples, representing fragments cut from the stem, were subjected to drying at room temperature.

### Convolutional neural networks method

**Fig. 2:**
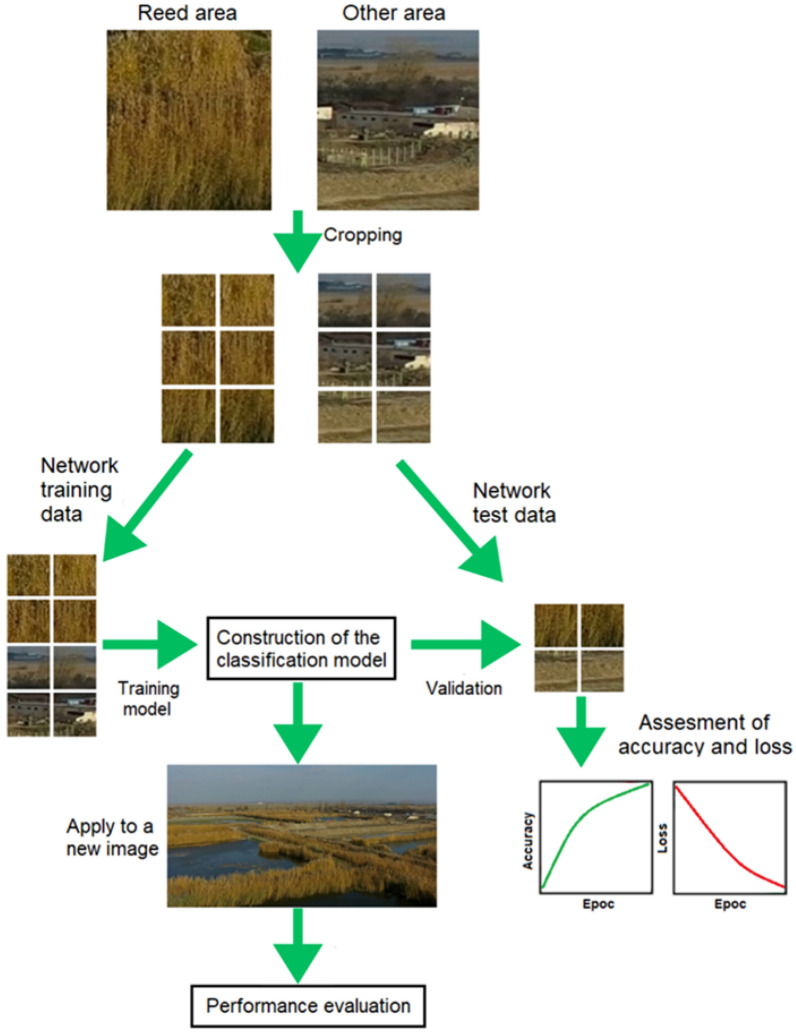
Outline of the approach adopted (images obtained using a drone)

In order to detect the reed in lake areas using artificial intelligence through neural networks using satellite images or obtained with the help of a drone, by recognizing the surfaces where reeds or rushes are present. In this study we propose CNNs (Convolutional Neural Networks) coupled with a particular type of feedforward neural networks composed of several convolution layers and grouping layers to detect reed from lake areas. To perform the convolutional neural network and the image pre-processing step the Matlab software package was used. This software is a MathWorks-developed numerical computing and statistical analysis development environment that contains the multi-paradigm programming language. Convolutional neural networks are composed of convolutional layers and pooling layers. Images are analysed using the convolutional layer to capture their features. In this process, a fixed-size filter runs over the images and extracts the patterns of shades of colours in the images. To reduce the variance of features, pooling layers follow each convolutional layer. These layers compute some operations on a particular feature over a region of the image to reduce its variance. Pooling layers serve two purposes. The pooling layer output amount does not change when the position of the feature amount extracted by the convolution layer shifts within the image, as the position sensitivity of the feature extracted by the convolution layer is reduced. Also, it expands the convolutional layers receptive field. An average and maximum operation can be performed on the pooling layers, this values are selected over the feature region. In this way, the pooling layer preserves the spatial location of the image, samples the output of the convolutional layer, and selects useful features for the following layer. All neurons in the previous layer are connected to every single neuron in the layer, after several convolutional and pooling operations. A classifier layer is then used to calculate a class probability for each image after all the convolutions, pooling, and fully connected layers have been applied. Furthermore, a probability calculation is conducted over every possible target class for each target class. A diagram of the proposed approach is shown in the Fig. 2. The training data of the network are developed using an image cropping method. First, in this method, images are collected in which the reed is 100% present and areas not covered with reed. Second, the obtained images are truncated into small squares, with an overlap of fifty percent both vertically and horizontally. Finally, the cropped images are used as training images. We created a model for classifying images using CNN to detect reed areas. In contradistinction to conventional approaches to form classifiers with hand-drawn feature extraction, CNN learns the hierarchy of features starting with pixels all the way to classifiers and trains common layers. The final layer of the CNN model is used to detect the coverage reed of images collected by a drone.

To avoid overlapping training and validation data, all small images are randomly shuffled, followed using 70% of the utilized images as training information and the other 30% as validation data. The network is built with two grouping layers, two convolution layers and one completely connected layer. The model uses several 30 training epochs. This method was validated during each learning era using two functions, such as accuracy and loss. “Accuracy” represents the precision of the method in classifying validation images, while “loss” is the inaccuracy of the model's prediction. If the model learning is successful, the accuracy is high, and the loss is low.

The evaluation of the performance of the proposed model is made by the confusion matrix. The results of the classification can be divided into the following four groups: false positive (FP), true positive (TP), true negative (TN) and false negative (FN).

### Characterization techniques

Morphological analysis of reed stems was performed by scanning electron microscopy (SEM). The dry reed was viewed under the electron microscope QUANTA 200 (FEI/ ThermoFischer Scientific), both the epidermis of the stem (straw) and in cross section. The samples were fixed on a metal support, by means of the double adhesive carbon tape. A vacuum pressure of 70 Pa and an electron acceleration voltage of 15 kV were selected for imaging analysis.

In this first stage of the chemical analyses, the following determinations were made: pH, conductivity, nitrate anion, nitrite anion, total nitrogen, sulphate anion, sulphide anion, phosphate anion. The analysis of total nitrogen, nitrate and nitrite concentrations were obtained with Cuvette test for Hach Lange DR5000 UV/VIS Spectrophotometer and the kit is expressed in g/L. The mentioned parameters were selected for analysis because two aspects were followed: first consists in their importance for the growth and development of the plant; and the second one is the analysis of the extract because, if some parameters have values that exceed the accepted norms, it will cause the pollution of the waters where the reeds grow.

The pH, conductivity and the salinity of the reed extract was determined with a multi-parameter analyser CONSORT C 533. The parameters mentioned before were measured in solution with weight ratio reed: distilled water 1: 5, decantation for 24 h and filtration.

The determination of the electrical conductivity of ionized mineral salts and the transformation of the electrical conductivity value into the content of electrical salts is done with the help of the conductometer. To determine the value of the constant of the conductometric cell (k), the electrical conductivity (c) is measured for two KCl potassium chloride solutions of 0.1n concentration; respectively 0.01 n.

The constant of the conductometric cell for the KCl 0.1n potassium chloride solution is calculated with the formula: k_1_ = γ_1_/c_1_, and for the solution of potassium chloride KCl 0.01n with the formula: k_2_ = γ_2_/c_2_ and the value of (k) will be equal to the value given by the formula [13]:

$$k=\frac{1}{2}\sum {k}_{i}$$

where γ_1_and γ_2_ are the specific conductivities (cm^−1^) of the two KCl solutions.

## Results and discussion

### Morphological characterisation

**Fig. 3:**
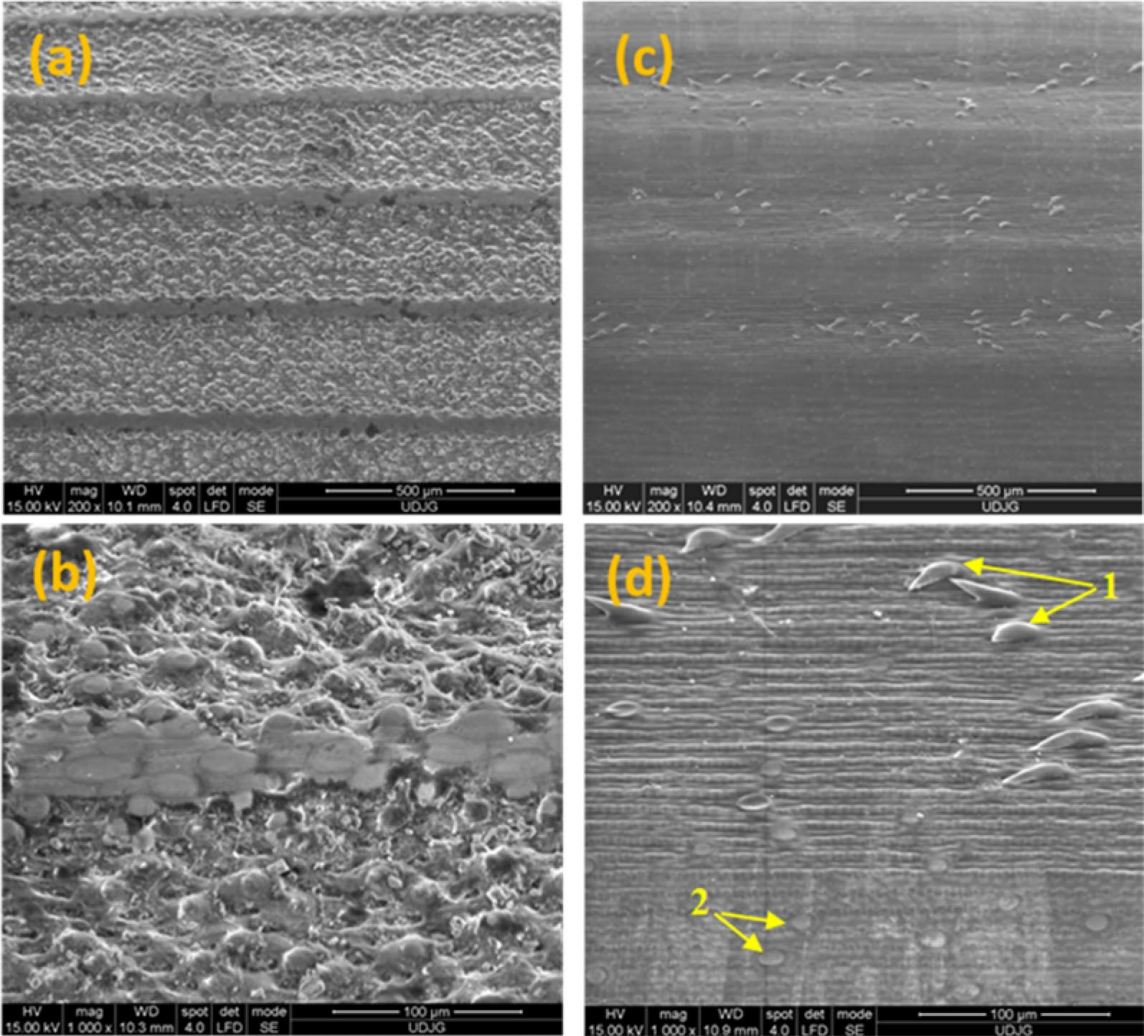
SEM images along the longitudinal direction of the reed stem epidermis: **a** and **b** control reed; **c** and **d** reed maintained in water (1. Non-glandular trichomes; 2. Glandular trichomes), at different magnifications (200 × and 1000x)

In Fig. 3 are indicated the microstructural aspects of the dry reed epidermis, along the longitudinal direction. A very high density of elliptical glandular structures can be seen on the surface of the control reed stem (Fig. 3a, b). In Fig. 3c, d, on the surface of the epidermis (cuticle) of the reed maintained in water, typical secretory structures can be observed, namely secretory glandular trichomes, respectively non-glandular trichomes. Microstructures on the outer surface indicate the presence of fibbers, tracheid and parenchyma cells, as anatomical elements of the reed stem.

**Fig. 4:**
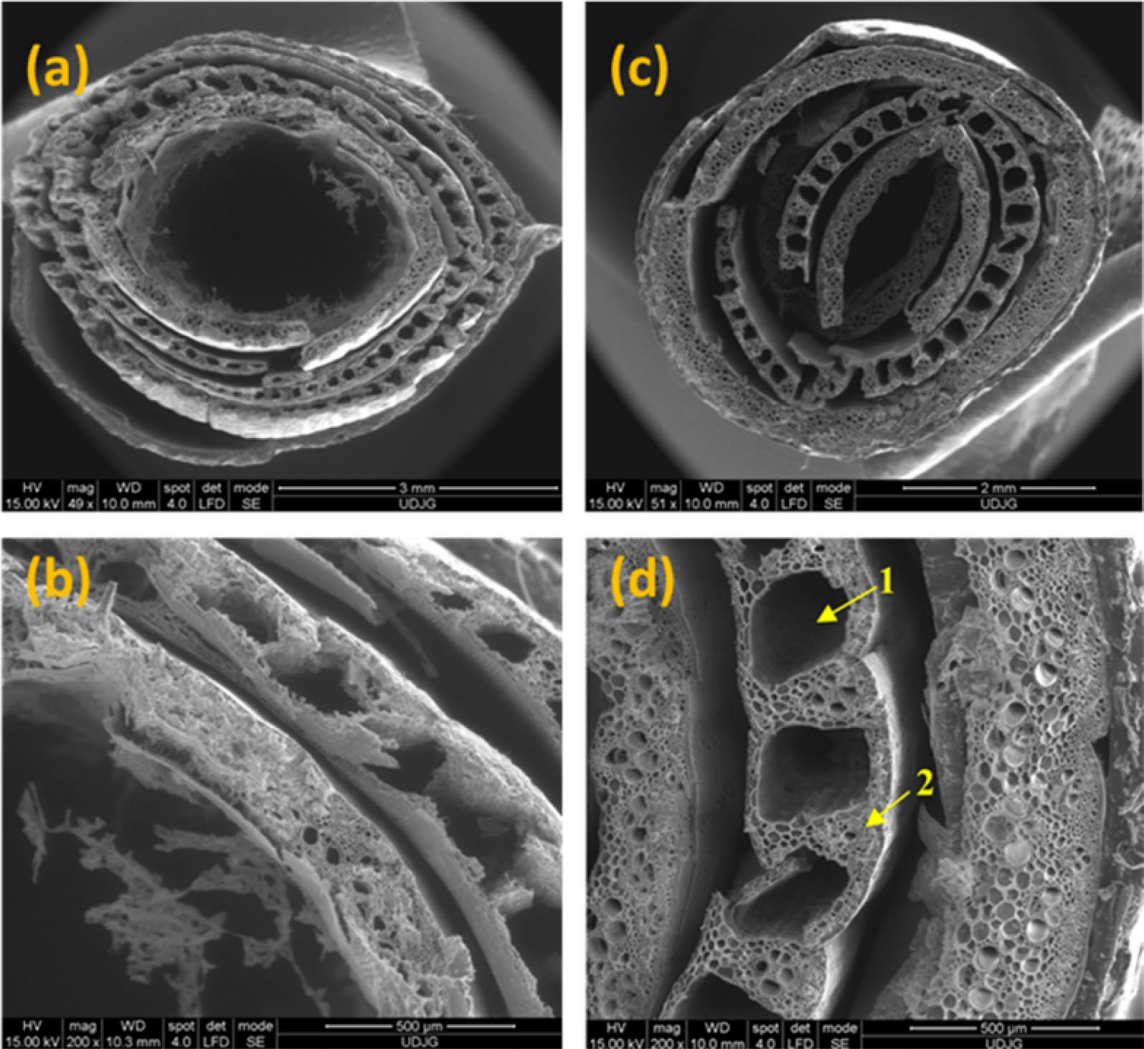
Cross-sectional SEM images of reed stem: **a** and **b** control reed; **c** and **d** reed kept in water, at different magnifications (50 × and 200x). Parenchymal tissue: 1. Air duct; 2. Conductive tissue

In Fig. 4 microstructural aspects of dry reed stalks are indicated in cross-section (total diameter of the section, 5 mm). Four concentrically arranged layers can be observed through the sectioned straw, the spongy character of the stems predominating.is predominated. The reed filters water well, the spongy structure of its stems contributes to the transport of oxygen to the root zones, enriching the soil, and having a beneficial effect on the development of other plant species and on the general state of the eco-system. The slightly elongated shape is due to plastic deformation, following mechanical cutting. On the outside, the cuticle has a tubular morphology, mainly with striations (fibbers), also visible in Fig. 4, which gives elasticity to the stem. Below the level of the epidermis, a homogeneous layer of angular cells can be observed that make up the collenchyma tissue. According to the shape of the isodiametric cells, the next inner cell layer is identified with the parenchymal tissue (thickness ~ 220 µm) and the spherical shape of the cells. Figure 4c, d correlate with Fig. 4c, d, indicating a decrease in the degree of roughness at the level of the epidermis, and water absorption causes a smooth appearance, an expansion of the sheath, which could activate a stem degradation mechanism. A major difference is found in the second cell layer, which shows structural changes in reed samples kept in water. These changes can induce severe effects on the rate of reed operation.

In Fig. 4b, d the typical structure of the parenchymal tissue showing air channels (1) and conductive tissues (2) was identified. This structure is more clearly high-lighted in the reed stalk kept in water for a long time. In the extensive intercellular air spaces of the parenchyma, called aerenchyma, quantities of air can accumulate, and are specific to aquatic plants. The role of these aerenchyma tissues is to prevent the penetration of fixing solutions into the plant tissues. The influence of water on the internal tissues of the reed stem is found in the degree of ordering and the irregular, elongated shape of the cells.

### Chemical analysis

The identification of chemical elements in the reed samples was performed by energy-efficient X-ray spectroscopy (EDX) and the results are showed in Table 1. The EDX spectra of the raw data of reed samples are presented in Additional File 1: Figure S1.

**Table 1:** Quantitative elemental chemical composition of the analysed reed samples: control reed (dry) and reed kept in water

Chemical elements (wt.%)	Sample type			
	control reed (epiderm)	Control reed (cross section)	Reed kept in water (epiderm)	Reed kept in water (cross section)
C	57.14	54.43	40.75	56.44
N	0.99	0.67	0.94	1.18
O	26.39	37.32	45.98	37.47
Na	0.13	0.09	0.11	0.10
Mg	0.09	0.05	0.07	0.00
Al	0.12	0.05	0.16	0.06
Si	13.77	2.83	10.74	1.96
P	0.02	0.00	0.09	0.15
Hg	0.00	0.26	0.26	0.00
S	0.00	0.04	0.00	0.17
Cl	0.09	0.55	0.05	0.24
K	0.15	0.77	0.09	0.55
Ca	0.08	0.51	0.00	0.54
Ti	0.10	0.00	0.00	0.00
V	0.10	0.00	0.00	0.00
Pb	0.10	0.49	0.12	0.00
Fe	0.41	0.80	0.19	0.63
Cr	0.00	0.71	0.24	0.51
Cu	0.34	0.42	0.19	0.00

From the elemental chemical analysis of the ash from the reed stalks, it follows that Fe, Cu, Cr, Al predominates among the metals, with variable amounts of Na, Mg, Si, K, V, Hg and Pb also present. Ash content is a measure of the presence of inorganic impurities in the common reed composition such as, or V, which can cause various types of problems for reed biomass applications. High concentrations of C (> 50 wt.%) and O (> 25 wt.%) indicate the presence of cellulose in reed straw.

Based on the results obtained from chemical analysis of the reed it can be concluded that the mineral combinations found in the reed stems include salts both in the form of water-soluble mineral substances (carbonates, sulphates, chlorides, oxalates) and insoluble ones (silicates, phosphates, calcium oxide, magnesium and manganese, ferric oxide).

Nutrients are components necessary for the growth and development of plants. It would be easy to consider a nutrient at a certain time, but it must be kept in mind that the plant needs nutrients holistically. Supplying one nutrient and ignoring others that the plant needs, including factors such as temperature, humidity, light, may have little benefit or may be to the detriment of the plants. Plants need 17 essential elements. Carbon, oxygen, and hydrogen are classified as non-mineral elements because they are taken from air and water. The other 14 essential elements represent soil nutrients and are divided into two categories: macronutrients and micronutrients [14, 15].

Macronutrients are necessary elements in fairly large quantities because they are used for the biological functions of plants such as growth, photosynthesis, and respiration. The category of macronutrients includes nitrogen, phosphorus, potassium, sulphur, calcium, and magnesium. Nitrogen is necessary for the growth of all plants. Phosphorus is the main component of many vital processes for plants. Potassium is necessary for a wide range of plant processes. Sulphur is necessary in the formation of amino acids, proteins, and vitamins or to produce chlorophyll. Calcium is useful in plant growth, especially roots. Magnesium is the essential component of chlorophyll and therefore vital for the photosynthesis process. Micronutrients, although needed in small amounts by plants, are just as important as macronutrients. If present in large amounts, micronutrients can be detrimental to plant growth. The category of micronutrients includes molybdenum, copper, boron, manganese, iron, zinc, nickel, and chlorine. Molybdenum is indirectly involved in nitrogen metabolism. Copper is necessary in the formation of enzymes to produce chlorophyll. Boron is used for the migration of sugars to plants and for nitrogen metabolism. Manganese, iron, and zinc are essential for the growth processes of plants. Nickel is the most recently identified nutrient for plants. It is the key component of nitrogen metabolism and its biological fixation. Chlorine is necessary in the metabolism of carbohydrates and in the production of chlorophyll [15–17] It can be considered a macronutrient because it is present in sufficient quantities in the environment and chlorine deficiency is rarely observed.

At the same time, the concentration of nutrients in the reeds are indicative for the decomposition of the biomass and the potential use of the reeds as ecological fuel [18–20].

#### Acid–base character (pH)

The pH influences the regime of micro and macro-elements. An acidic pH indicates very low calcium values or sometimes this element is missing even though it is vital for plant life. Deficiencies of microelements (boron, molybdenum, cobalt, etc.) are also found at acidic pH due to intense leaching or blocking of these elements in the form of compounds inaccessible to plants. An extract with an acid reaction contains significant amounts of iron, aluminium, and manganese, which are toxic for plant development. The accessibility of phosphorus for plants is influenced by the acid–base character of the extract. At pH = 5–7 phosphates are soluble and phosphorus is accessible to plants. If the pH drops below 5, phosphorus is found in the form of iron and aluminium phosphates, which are insoluble, and plants cannot use the phosphorus [21, 22].

At alkaline pH, phosphorus is in the form of tricalcium phosphate, hardly soluble, hardly accessible to plants. The alkaline reaction is also harmful, the plants not being able to develop at a pH higher than 8.5. Alkalinity leads to the blocking of some microelements (Cu, Zn, Mo, B, etc.), and large amounts of sodium determine unfavourable physical properties.

#### Electrical conductivity. The amount of soluble salts

The electrical conductivity is given by the salts that dissociate in the aqueous extract. The total amount of salts in the extract is determined by the soluble salts stored in the reed that feed the plant with anions and cations necessary for the growth and development of the plant. Many soluble salts in the extract would determine the pronounced alkalinity of the extract, the plant would have an excess of nutrients that will leach into the water, then into the reed, leading to salinity and salinity of the reed and its infertility [23]. Table 2 shows the values of pH, conductivity and content of soluble salts obtained for the reed extracts.

**Table 2:** The values of pH, conductivity, total amount of salts for reed extracts

Sample	pH, pH units	Electricity conductivity, mS/cm	The amount of soluble salts, g/L
1	4.98	5.63	3.24
2	4.94	2.69	4.70
3	4.91	2.31	4.07

From the data presented, it can be observed that the three samples have a moderately acidic pH, with values in the range of 4.91–4.98 pH units. The number of soluble salts in the reed extract is in the range of 3.24–4.70 g/L, the values are within normal limits [24, 25], providing the plant with the necessary nutrients.

#### Total nitrogen. Nitrates. Nitrite

Plants take nitrogen from the reed in the form of nitrate anions or ammonium cations. Most organic compounds in plants contain nitrogen, including amino acids, nucleic acids, enzymes, and energy-transferring substances such as chlorophyll, adenosine triphosphate (ATP) and adenosine diphosphate (ADP). Plant growth is achieved with the help of nitrogen, which is necessary in the formation of new cells, essential for plants. Nitrogen has a plastic role for plants, it is found in all proteins, influences crop yield and is the main constituent of the cytoplasm.

Nitrogen deficiency symptoms for plants are manifested by slow plant growth, poor branching, low protein content of crops, burnt appearance of leaves, yellowing or chlorosis of leaves. In severe nitrogen deficiencies, the leaves turn brown and die [13, 14].

Excess nitrogen in plants is manifested by dark green colour, vegetative growth at the expense of seed production, increased sugar content, delayed flowering and fruiting, boron and copper deficiency due to the inhibition of the adsorption of these nutrients by nitrogen.

**Fig. 5:**
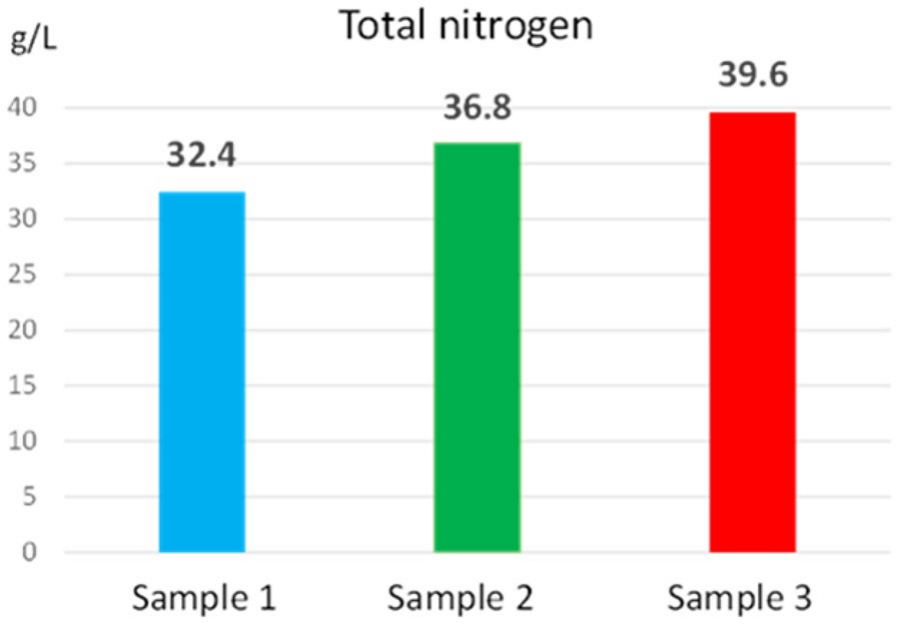
Total nitrogen concentration determined from reed extract

**Fig. 6:**
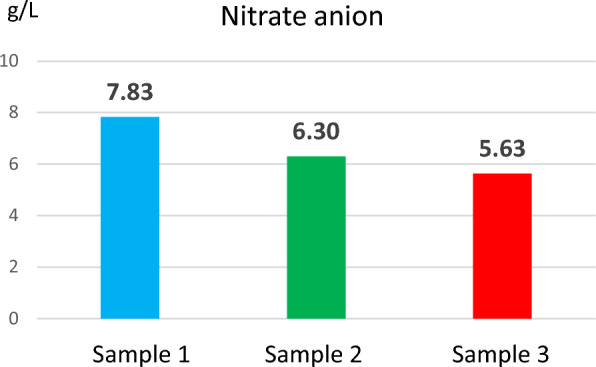
Nitrate anion concentration determined from reed extract

**Fig.7:**
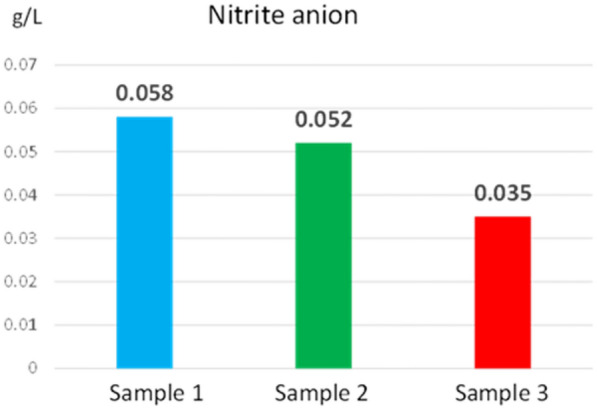
Nitrite anion concentration determined from reed extract

Figures 5, 6, 7 show the concentrations determined for total nitrogen, nitrate anion and nitrite anion from the reed extracts.

The concentration values of total nitrogen (32.4–39.6 g/L), nitrate anion (7.83–5.63 g/L) and nitrite anion (0.058–0.035 g/L) ensure the nitrogen requirement for growth and development the plant. At the same time, these values are within the limits of the allowed values for water [24], which proves that reeds do not negatively influence the ecosystem.

Nitrogen in plant tissue is mainly a component of proteins and other easily degradable compounds. High nitrogen concentration is generally found in the leaves, but the nitrogen concentrations in the samples analysed were comparably low. Low nitrogen could pose a challenge for biomethane production or biomass harvesting for nutrient removal. Biomass produced earlier in the growing season is usually richer in nitrogen than late in the season. The production of biomethane to be more efficient, it is necessary to choose the optimal season for harvesting the plant, so that the nitrogen in the plant has an optimal concentration. It must also be considered that, if the biomass is intended to be used as a solid fuel, the nitrogen content of the plant would be useful to be low because the ash concentration is reduced.

#### Sulphate anion. Sulphide anion

Sulphur is the component of three important amino acids (cystine, cysteine and methionine) incorporated in proteins. Sulphur is essential in the formation of chlorophyll and in the activation of some enzymes. It is also important for the formation of nodules by nitrogen-fixing bacteria in vegetable roots. It is found in biotin and thiamine (vitamin B1) and is necessary for the formation of some oils from mustard plants, some hydrogen bonds existing in onions, garlic and in various oils. Sulphur is necessary for plants in quantities comparable to phosphorus [24, 25]. The intake between N_total_/S_total_ is about 15, and the normal amount of sulphur in plant tissue is 0.12–0.35%.

Sulphur deficiency could occur if fertilizers based only on N, P, K without S are used. Sulphur deficiency symptoms include: paleness of young leaves; the colour of the limb and veins becomes yellowish green; the yellow colour is not as pronounced as in the case of nitrogen deficiency; the veins of the leaves, especially in the upper part, sometimes acquire a lighter colour compared to the neighbouring tissues; sulphur-deficient plants are small and frail with short and fragile stems; plant growth is slowed down and maturation is delayed; sulphur deficiency affects the accessibility of molybdenum, an essential element in the biological fixation of nitrogen [26, 27].

The reed roots extend up to approximately 95 cm in the soil, where the water and nutrient requirements are ensured, and the extensive network of rhizomes ensures the storage of substances in the cold season and the asexual growth of the plant even if the above-ground stems are cut [28–30].

Sulphur is adsorbed by plants from the sulphate anion. Sometimes it can be adsorbed from the air through leaves in industrial areas where sulphur is in high concentrations. The use of organic residues in satisfactory quantities for the needs of other nutrients will also ensure the quantities of sulphur necessary for the normal development of plants [31].

Under normal conditions of pH and redox potential of the soil, the stable form of sulphur is SO_4_^2−^, an anion easily transported and assimilated by plants. Reduction of SO_4_^2−^ anion to H_2_S occurs at redox potential around − 100 mV and pH 6. The direct impact of pH and redox potential (EH) on sulphur solubility is limited to waterlogged soils or submerged fields in water. In these soils, especially when they are rich in organic matter, a low redox potential causes the transformation of SO_4_^2−^ into H_2_S which is very toxic to plants. At high values of EH and pH the availability of sulphur is affected indirectly because EH and pH influence the adsorption capacity of sulphur by the soil. In natural environments, sulphur is one of the main determinants of EH–pH characteristics.

Excess sulphur can cause, under strong reducing conditions, large amounts of hydrogen sulphide, which is very toxic. Since sulphur can also be adsorbed from the atmosphere in the form of SO_2_, concentrations higher than 0.6 g/m^3^ of SO_2_ are toxic to plants (the maximum concentration is 0.1–0.2 g/m^3^ SO_2_). Sulphur toxicity is manifested by necrotic spots on the leaves, which then spread over the entire surface of the leaf blade.

**Fig. 8:**
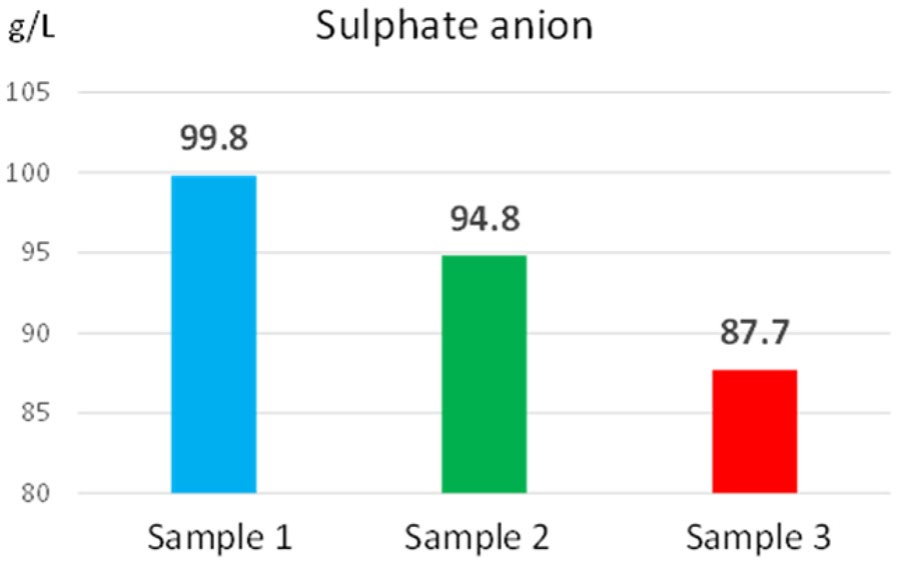
Sulphate anion concentration determined from reed extract

**Fig. 9:**
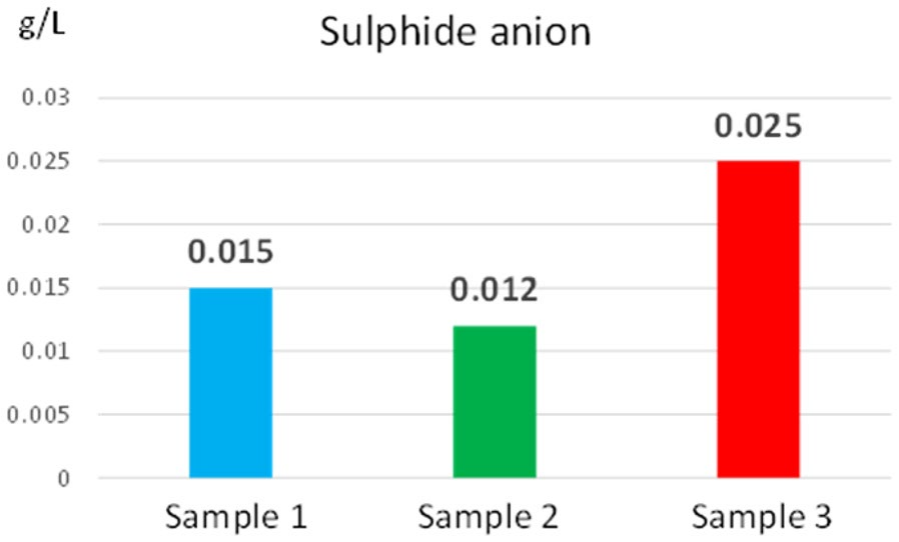
Sulphide anion concentration determined from reed extract

The concentration values of sulphate (87.7–99.8 g/L) and sulphide (0.012– 0.025 g/L) anions determined from the three reed extracts are shown in Figs. 8, 9. The concentration values of the sulphide anion and the sulphate anion ensure the amount of sulphur needed by the reed to grow and develop.

At the same time, the low concentration of the sulphide anion does not lead to the formation of hydrogen sulphide, which would greatly increase the acidity of the water and influence the ecosystem. Like nitrogen, if biomass is to be used as a solid biofuel, the sulphur concentration must be minimal to reduce the ash concentration.

#### Phosphate anion

Phosphorus is present in all living cells as part of deoxyribonucleic acid and ribonucleic acid, component of phospholipids in cell membranes and in molecules for energy storage and transfer (adenosine triphosphate). Limits plant growth and root formation, speeds up the flowering period, stimulates production and seed size. It is used in the synthesis of proteins and is found in the nodules of legumes. The amount of phosphorus in the plant and in the reed must be in balance with the amount of nitrogen. Since nitrogen is more mobile and phosphorus is one of the nutrients with the lowest mobility, excessive availability of nitrogen can cause phosphorus deficiency [29, 31].

**Fig. 10:**
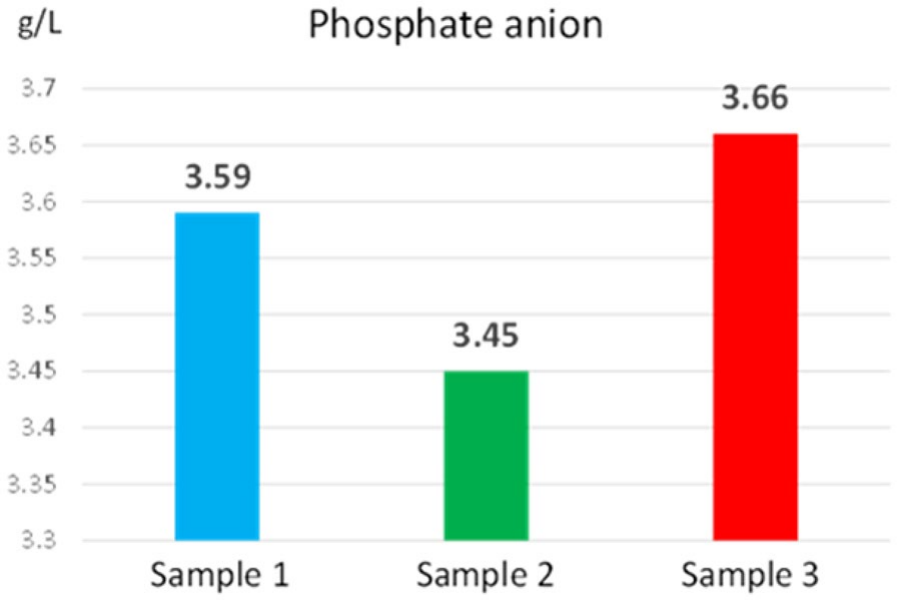
Phosphate anion concentration determined from reed extract

Phosphorus deficiency is manifested by slow growth, blackening of plants, purple colour on the leaves of some plants, dark green colour at the tips of dead leaves, delayed ripening, poor development of grains, fruits or seeds. Insufficiency of phosphorus in chloroplasts reduces the process of photosynthesis. Due to the deficiency of phosphorus in plants, the synthesis of deoxyribonucleic acid is reduced, and protein synthesis is also reduced. Excess phosphorus in the reed can cause symptoms of toxicity that are manifested by watery edges of the leaf tissues, and over time they can become necrotic. In severe cases of excess phosphorus, plant death may occur. The excess of this nutrient induces secondary zinc deficiencies. Phosphorus is the macronutrient with very low mobility. Since the roots can only take it up if it is within a few centimetres of them, the source of phosphorus must be close, otherwise it is not available [32]. Maintaining adequate moisture throughout the growing season facilitates the movement of phosphorus into the reed. The availability of phosphorus in the reed is also affected by temperature. At low temperatures, the plants register a phosphorus deficiency even if there are enough in the reed. As the temperature rises, the phosphorus deficiency symptoms begin to disappear. The phosphate anion concentration values (3.45–3.66 g/L) determined from the three reed extracts are shown in Fig. 10.

Based on the results of chemical analysis of the reed extract we can affirm that biomass harvested during winter will have weaker correlations between minerals but constant morphological parameters because nutrient concentrations in aboveground biomass change during seasons. Harvesting reeds for bioenergy is therefore a sustainable way to obtain biomass, as reeds are a renewable underground biomass resource with valuable energetic properties. The present research will be continued with possibilities of re-use of the harvested reed in accordance with AGENDA 2030 for Sustainable Development.

## Conclusions

This is the first time that the performance of the convolutional neural network and the image pre-processing step are used to detect and predict the areas at risk for biodiversity (reduction of water gloss until it disappears, imbalances caused by keeping reeds dry in water) caused by the aggressive and uncontrolled growth of reeds. The morphological results of the study indicate microstructures on the outer surface indicate the presence of fibbers, tracheid and parenchyma cells, as anatomical elements of the reed stem.

The mineral combinations found in the reed stems include salts both in the form of water-soluble mineral substances (carbonates, sulphates, chlorides, oxalates) and insoluble ones (silicates, phosphates, calcium oxide, magnesium and manganese, ferric oxide). Nitrogen, sulphur, and phosphorus from plants are mostly assimilated as anions, and Ca and Na are assimilated as cations. This fact ensures the synergy of ions which is very pronounced between anions and cations, resulting in the tendency of the electrochemical balance between the two species of ions. Thus, the high absorption of the nitrogen anion leads to the high absorption of calcium to maintain the chemical balance.

The concentration of nitrogen and phosphorus must be correlated because the increase of one nutrient leads to the decrease of the other, and the plant needs a balance between the concentrations of nitrogen and phosphorus for normal growth and development.

Biomass harvested during winter will have weaker correlations between minerals but constant morphological parameters because nutrient concentrations in aboveground biomass change during seasons. Harvesting reeds for bioenergy is therefore a sustainable way to obtain biomass, as reeds are a renewable underground biomass resource.

From the goal of this research, we are setting out a supremely ambitious and transformational vision by finding new opportunities to sustainable recycling and safe reuse globally of the dried reed.

## Supplementary Information

**Additional file 1: Figure S1:** EDX spectra – qualitative chemical analysis of the analysed reed samples: a) and b) control reed (epidermis and cross-section); c) and d) reed maintained in water (epidermis and cross section). **Figure S2:** EDX mapping of the analysed reed samples.

## Data Availability

All data analysed during this study are included in this published article and its supplementary information files.

## References

[1] Haslam SM (2010). A book of reed: Phragmites australis (Cav) Trin ex Steudel Formerly Phragmites communis Trin.

[2] Sweers W, Horn S, Grenzdörffer G, Müller J (2013). Regulation of reed (Phragmites australis) by water buffalo grazing: use in coastal conservation. Mires Peat.

[3] Wichtmann W, Couwenberg J (2013). Reed as a renewable resource and other aspects of paludiculture. Mires Peat.

[4] Kroon FW (2013). Saving reed lands by giving economic value to reed. Mires Peat.

[5] Hiss Reet Dachreet (Thatching reed). Online at Accessed from 22 February 2023.

[6] Li J, Zhang L, Huang G, Wang H, Jiang Y (2021). Experimental study on creep properties prediction of reed bales based on SVR and MLP. Plant Methods.

[7] FNR (2012). Dämmstoffe aus nachwachsenden Rohstoffen (Insulation from Renewable Resources) Fachagentur für nachwachsende Rohstoffe (FNR).

[8] Bessou C, Ferchaud F, Gabrielle B, Mary B (2011). Biofuels, greenhouse gases and climate change. A Review. Agronomy Sust Developm.

[9] Suárez L, Barczewski M, Kosmela P, Marrero MD, Ortega Z (2023). Giant Reed (Arundo donax L.) fiber extraction and characterization for its use in polymer composites. J Nat Fibers..

[10] Idupulapati NB. A Theoretical Study of Methanol Oxidation Mechanism by Methanol Dehydrogenase Enzymes for Fuel Cell Applications. Ph.D. Thesis, Louisiana Tech University United States, Ruston, LA, USA, 2009.

[11] Catana RD, Podosu A, Florescu LI, Mihai RA, Enache M, Cojoc R, Moldoveanu M (2023). Quantitative analyses of chemical elements in phragmites australis as bioindication of anthropization in Urban Lakes. Sustainability.

[12] Government Decision no. 971 of 05.10.2011 for the amendment and completion of Government Decision no. 1,284/2007 regarding the declaration of areas of special avifaunistic protection as an integral part of the European Natura 2000 ecological network in Romania; Accessed from 20 February 2023.

[13] Sposito G (1989). The chemistry of soils.

[14] Barker AV, Pilbeam DJ (2007). Handbook of Plant Nutrition.

[15] Konrad M, Kirkby E, Kosegarten H, Appel T. Principles of Plant Nutrition, 5th Edition, Kluwer Academic Publishers. 2001.

[16] Marschner P (2012). Marschner's Mineral Nutrition of Higher Plants.

[17] Barker AV, Pilbeam DJ (2007). Handbook of Plant Nutrition.

[18] Ren L, Eller F, Lambertini C, Guo WY, Brix H, Sorrell BK (2019). Assessing nutrient responses and biomass quality for selection of appropriate paludiculture crops. Sci Total Environ.

[19] Geurts JJM, Oehmke C, Lambertini C, Eller F, Sorrell BK, Mandiola SR, Grootjans AP, Brix H, Wichtmann W, Lamers L (2020). Nutrient removal potential and biomass production by Phragmites australis and Typha latifolia on European rewetted peat and mineral soils. Sci Total Environ.

[20] Ding Y, Wang Y, Gu X, Peng Y, Sun S, He S (2023). Salinity effect on denitrification efficiency with reed biomass addition in salt marsh wetlands, Bioresour. Technol.

[21] Bradová M, Tejnecký V, Boruvka L, Němeček K, Ash C, Šebek O, Svoboda M, Zenáhlíková J, Drábek O (2015). The variations of aluminium species in mountainous forest soils and its implications to soil acidification. Environ Sci Pollution Res.

[22] Essington ME (2015). Soil and Water chemistry: an integrative approach.

[23] Smith JL, Doran JW, Doran JW, Jones AJ (1996). Measurement and Use of pH and Electrical Conductivity for Soil Quality Analysis. Methods for assessing soil quality, soil science society american special, 49, SSSA.

[24] SR EN 26777: 2006. Water quality. Determination of nitrite content. Method by molecular absorption spectrometry.

[25] Calmuc M, Calmuc V, Arseni M, Topa C, Timofti M, Georgescu LP, Iticescu C (2020). A Comparative approach to a series of physico-chemical quality indices used in assessing water quality in the lower Danube. Water.

[26] Sparks DL (2001). Elucidating the fundamental chemistry of soils: past and recent achievements and future frontiers. Geoderma.

[27] Husson O (2013). Redox potential (Eh) and pH as drivers of soil/plant/microorganism systems: a transdisciplinary overview pointing to integrative opportunities for agronomy. Plant Soil.

[28] Calmuc VA, Calmuc M, Arseni M, Topa CM, Timofti M, Burada A, Iticescu C, Georgescu LP (2021). Assessment of heavy metal pollution levels in sediments and of ecological risk by quality indices, applying a case study: the lower danube river. Romania Water.

[29] Hinsinger P, Plassard C, Tang C, Jaillard B (2003). Origins of root-mediated pH changes in the rhizosphere and their responses to environmental constraints: a review. Plant Soil.

[30] Eller F, Guo X, Ye S, Mozdzer TJ, Brix H (2020). Suitability of wild phragmites australis as bio-resource: tissue quality and morphology of populations from three continents. Resources.

[31] Brady NC, Weil RR (2008). The Nature and Properties of Soil, 14th Edition, Pearson Prentice Hall.

[32] Goldberg S, Sposito G (1985). On the mechanism of specific phosphate adsorption by hydroxylated mineral surfaces: a review. Commun Soil Sci Plant Anal.

