# Supplementary material for: Detection of reed using cnn method and analysis of the dry reed (phragmites australis) for a sustainable lake area
Source: Plant Methods. 2023 Jun 24;19:61. doi: 10.1186/s13007-023-01042-w (PMC10290358; doi:10.1186/s13007-023-01042-w)
Supplement: Supplementary file 1 — Additional file 1: Figure S1: EDX spectra – qualitative chemical analysis of the analysed reed samples: a) and b) control reed (epidermis and cross-section); c) and d) reed maintained in water (epidermis and cross section). Figure S2: EDX mapping of the analysed reed samples. [file 13007_2023_1042_MOESM1_ESM.docx]

**DETECTION OF REED USING CNN METHOD AND ANALYSIS OF THE DRY REED (PHRAGMITES AUSTRALIS) FOR A SUSTAINABLE LAKE AREA**

Cristian Dragos Obreja^1^, Daniela Laura Buruiana^1^, Elena Mereuta^2^, Alina Muresan^1^, Alina Mihaela Ceoromila^3^, Viorica Ghisman ^1,*^ and Roxana Elena Axente^4^

^1^ Interdisciplinary Research Centre in the Field of Eco-Nano Technology and Advance materials CC-ITI, Faculty of Engineering, “Dunarea de Jos” University of Galati, Romania.

^2^ Dunarea de Jos Univ Galati, Dept Mech Engn, 47 Domneasca St, Galati 800008, Romania.

^3^ Research and Development Center for Thermoset Matrix Composites, Cross-Border Faculty ”Dunarea de Jos” University of Galati, Romania.

^4^ Medicine and Pharmacy Faculty, “Dunarea de Jos” University of Galati, 47 Domneasca, 800008 Galati, Romania

*Correspondence to*: Viorica Ghisman ([viorica.ghisman@ugal.ro](mailto:Daniela.buruiana@ugal.ro))

The EDX spectra of the raw data of reed samples are presented in Fig. S1 recorded over the entire surface of the analysed area and EDX mapping of the analysed reed samples is presented in Fig. S2.

*
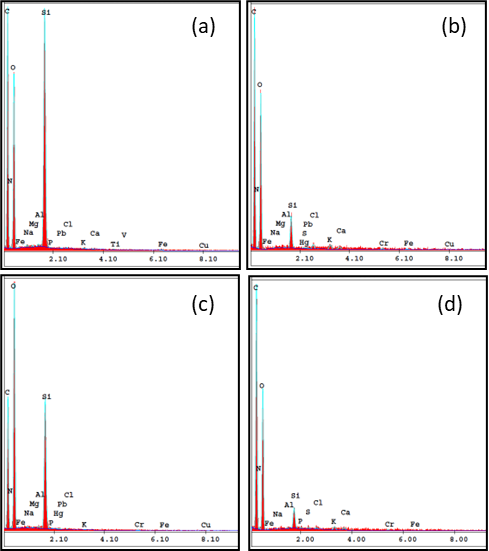
*

**Figure S1:** EDX spectra – qualitative chemical analysis of the analysed reed samples: a) and b) control reed (epidermis and cross-section); c) and d) reed maintained in water (epidermis and cross section).

**Figure S2:** EDX mapping of the analysed reed samples.
